# Management of twenty centimeter segmental bone defect of femoral shaft secondary to infected non-union of fracture using masquelet technique: A case report

**DOI:** 10.1016/j.ijscr.2021.106107

**Published:** 2021-06-10

**Authors:** Kyle Kubes, Alex Friedman, Casey Pyle, Graal Diaz, Damayea Hargett

**Affiliations:** a Community Memorial Hospital, Ventura, CA, United States of America; b Ventura County Medical Center, Ventura, CA, United States of America

**Keywords:** Case report, Limb salvage, Non-Union of Fracture, Masquelet technique, Large bone defect

## Abstract

**Introduction:**

Segmental bone loss is a challenging condition to manage, and some of the techniques employed are difficult for patients to tolerate and involve lengthy treatment and rehabilitation times. The Masquelet technique is a two-stage bone grafting technique used to treat segmental bone defects. The technique has primarily been described for bone defects averaging 5.5 cm in length. This technique's advantages include protection against autograft resorption, relative maintenance of graft position, and prevention of soft-tissue interposition. We present a case report of a male who achieved successful bone defect union utilizing the Masquelet technique for a right femoral shaft infected non-union with a resultant 20 cm bone defect.

**Case report:**

This is a case report of a 28-year old male who presented to our clinic for evaluation and treatment for a segmental bone defect secondary to a right femur fracture with non-union after infection. The patient had been in a motor vehicle collision. Our patient was interested in limb salvage surgery and declined bone transport. Given the significant size of his defect, we opted to treat him utilizing the Masquelet technique. He went on to have a successful union of his defect with associated increased subjective quality of life and functionality.

**Conclusion:**

The Masquelet technique is a useful limb salvage treatment for patients with segmental bone defects, including large defects of 20 cm in length.

## Highlights

• Limb salvage surgery
• Use of Masquelet technique for a large bone defect
• Successful treatment of a 20 cm femoral shaft bone defect secondary to trauma and subsequent infection.

## 1 Introduction

Segmental bone loss due to trauma and subsequent infection is a challenging condition to manage. Options for limb salvage management include autogenous bone grafting, free vascularized fibular bone grafts, bone transport with distraction osteogenesis, and induced membrane technique. Some of the techniques employed are difficult for patients to tolerate and involve lengthy treatment and rehabilitation times [1].

The induced membrane technique, also known as the Masquelet technique, is a two-stage bone grafting technique used to treat segmental bone defects and non-unions. The technique was first presented by Masquelet in 1986, publishing a case series fourteen years later [2]. This technique's advantages include protection against autograft resorption, relative maintenance of graft position, and prevention of soft-tissue interposition [1,3].

The first step involves the initial debridement of soft tissue and bone down to healthy, bleeding tissue. This is followed by using a polymethylmethacrylate (PMMA) cement spacer (with or without antibiotics) placed into the bony defect, which is stabilized using either internal or external fixation. The spacer's role is to prevent fibrous ingrowth into the bone defect and induce an inflammatory reaction that results in the development of a surrounding pseudo-synovial membrane [1,3]. Histologic and immunochemical analysis of the induced membrane in animal models has demonstrated extensive vascularization of the membrane and production of transforming growth factor-beta (TGF-beta) and vascular endothelial growth factor (VEGF), as well as the osteoinductive factor bone morphogenetic protein-2 (BMP-2), with the highest levels of BMP-2 recorded at four weeks after implantation of the spacer [4].

The second step occurs 4–8 weeks later and involves incision of the membrane and removing the spacer. Bone graft is then placed into the cavity, followed by definitive closure of the membrane and fracture fixation. Various methods of bone grafting have been described, including Reamer-Irrigator-Aspirator (RIA), iliac crest, allograft, bone substitutes, demineralized bone matrix/bovine bone, growth factors, and vascularized or non-vascularized fibular autograft [1,3]. Recorticalization generally occurs by 3 to 6 months [1,3].

The Masquelet technique has been described primarily for bone defects averaging 5.53 cm in length; however, there are few reports in the literature regarding its use in bone defects 20 cm or greater [2,5,6]. We present a case describing a successful treatment of a 20 cm femoral shaft bone defect secondary to trauma and subsequent infection. The patient was informed that data concerning the case would be submitted for publication, and he provided consent.

## 2 Case presentation

We present a case report of a 28-year old male who presented to our clinic for evaluation and treatment for a segmental bone defect secondary to a right femur fracture with non-union after infection. The patient had been in a motor vehicle collision in his home country of Uganda approximately two years prior to presentation at our clinic. He had sustained an isolated, closed, mid-shaft right femur fracture and was treated at the time of injury with an intramedullary (IM) femoral nail. Unfortunately, his medical records, injury, and initial post-operative radiographs from Uganda were unavailable for this report. The patient was in good health at the time of injury and was employed as an attorney.

**Fig. 1:**
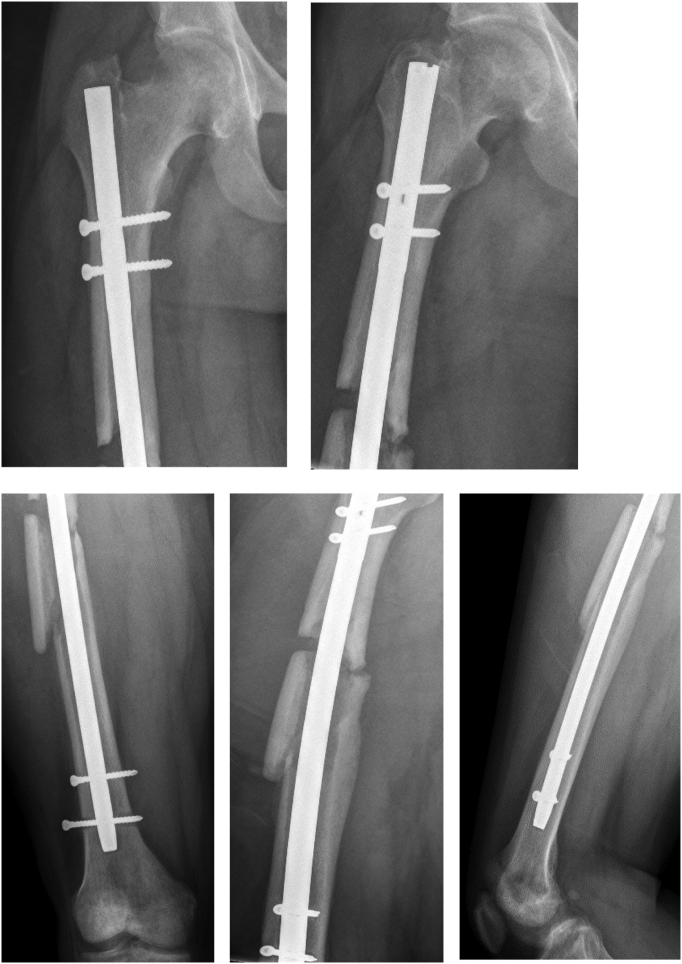
Patient's radiographs upon initial presentation for his infection in the US with original IM nail from Uganda in place.

Approximately three months after his initial surgery, he was noted to have signs and symptoms of an infected non-union, and underwent irrigation & debridement (I&D) in Uganda. The patient subsequently immigrated to the United States and presented at another institution with a draining sinus tract at his fracture site. He was placed on multiple antibiotics and underwent an additional I&D. His original IM nail was left in place at that time [Fig. 1]. The patient's intraoperative cultures grew a multi-drug resistant species of enterococcus and a methicillin-sensitive staphylococcus. Over the next twelve months, the patient was treated with multiple antibiotics and underwent five additional I&Ds, the latter four including additional bone excision and attempts at induced membrane technique with intramedullary nail exchanges and antibiotic cement spacer placement. Prior to his final surgery, skin wound cultures grew a multi-drug resistant *Staphylococcus epidermidis* species.

**Fig. 2:**
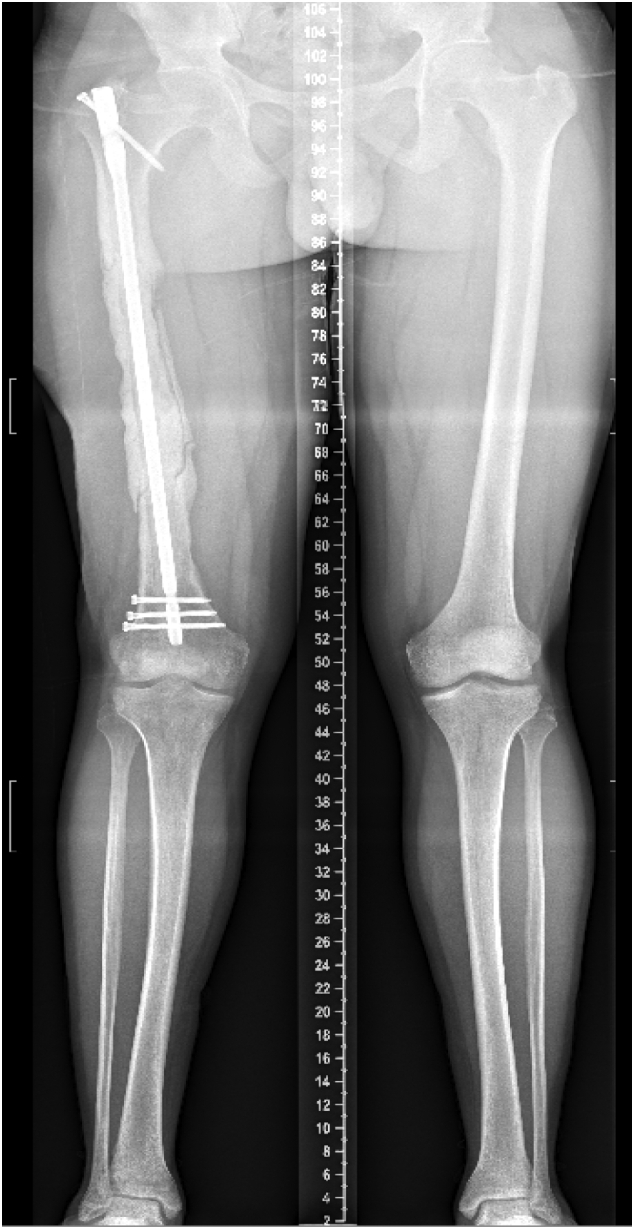
Patient's radiographs upon presentation to our clinic demonstrating a 20 cm segmental defect of his right mid-shaft femur with intramedullary nail and cement spacer in place.

**Fig. 3:**
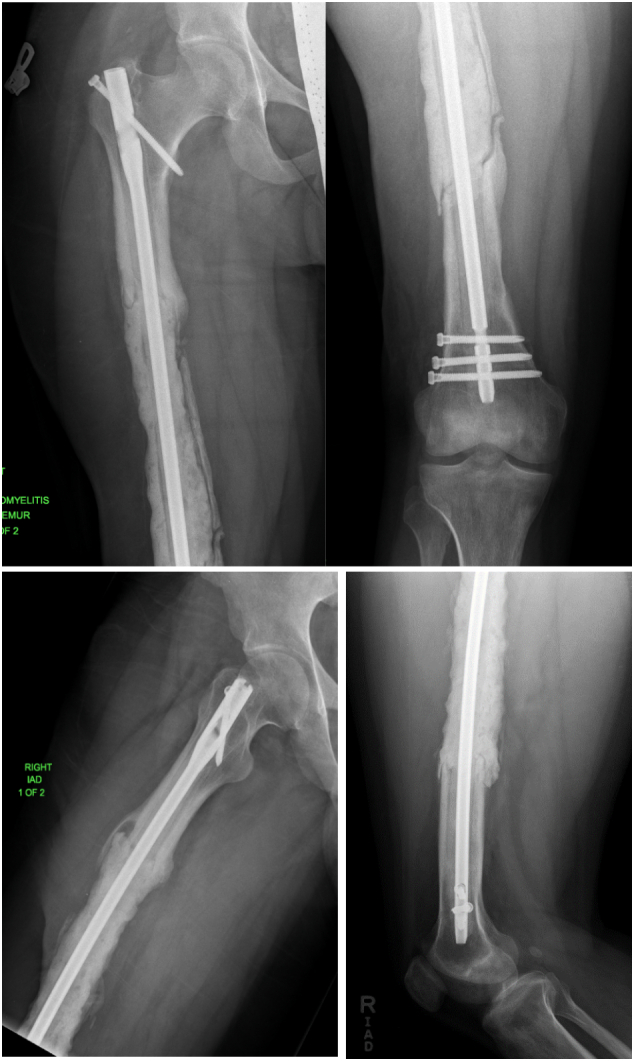
Patient's radiographs upon presentation to our clinic demonstrating a 20 cm segmental defect of his right mid-shaft femur with intramedullary nail and cement spacer in place.

At the time of presentation to our clinic, the patient was three months post-operative from his last antibiotic nail exchange, with an eight mm antibiotic coated intramedullary nail (Smith and Nephew; London, UK) in place, and was no longer taking antibiotics. Radiographs showed a 20 cm bone defect at the mid-shaft of the right femur, as well as a 3 cm leg length discrepancy [Fig. 2, Fig. 3].

After consultation with our infectious disease specialist, along with a thorough laboratory infection workup, it was presumed that the infection had been eradicated. The patient also underwent a thorough medical, endocrine and metabolic workup to ensure no other conditions contributed to the non-union. He was optimized for surgical treatment of his bone defect.

His prior Masquelet attempt was three months prior, beyond the four-week time point when the pseudomembrane's biologic activity was at its highest [4]. We determined that repeating the first stage of the Masquelet procedure would optimize the chances of success and allow for confirmation through cultures and tissue specimens that there was no residual infection. A thorough discussion took place with the patient regarding treatment options, and he opted to proceed with surgical treatment utilizing the induced membrane technique.

The first stage of his Masquelet operation was then performed. The patient was placed on a fracture table, and a lateral incision was carried out through the patient's old scar to access the femoral bone defect as well as his cement spacer. The patient had abundant scar tissue, and large full-thickness flaps were elevated to expose the spacer. Because of the patient's multiple attempts at previous Masquelet procedures, he had a robust membrane already formed that was incised to gain access to this space. The previously placed antibiotic cement spacer, followed by the intramedullary nail, was removed. Multiple tissue samples were sent for frozen section and culture, which were found to be without evidence of infection (<5 WBC/HPF). The membrane cavity was then debrided of all scar and fibrinous tissue, and proximal and distal bone ends were bleeding and appeared healthy. Next, the Reamer Irrigator Aspirator (RIA; DepuySynthes; Johnson & Johnson Co. Inc., NJ) was used to debride the femoral canal using 3 l of normal saline (NS) with bacitracin, followed by 3 l of NS. Given the patient had a leg length discrepancy and a mild external rotational deformity, traction and five degrees of internal rotation were applied to attempt to improve limb length and rotational alignment.

A new lateral entry antegrade femoral intramedullary nail (12x440mm; DePuy Synthes, Raynham, Massachusetts) with one proximal and three distal interlocking screws was placed. Next, a temporary PMMA (Palacos; Zimmer, Warsaw, IN) antibiotic cement spacer was made by mixing three bags (40 g per bag) of cement, 7 g vancomycin, and 5 g of meropenem. These antibiotics were chosen based on the patient's known pathogen history. The cement was placed around the femoral nail during the later stages of polymerization to better shape the cement and prevent interdigitation of the cement to adjacent bone. Once the cement hardened, the entire wound was copiously irrigated, and the membrane and soft tissues were closed in a layered fashion. A hemovac drain was placed superficial to the fascia.

**Fig. 4:**
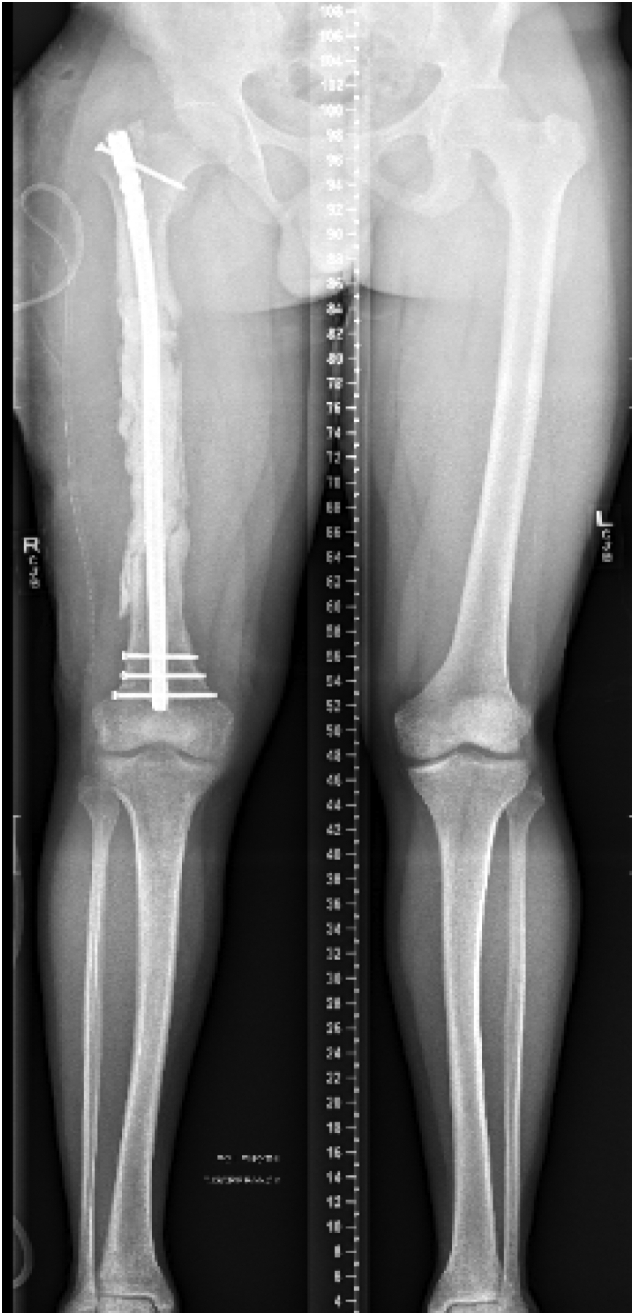
Patient's standing alignment radiograph after the first stage of his Masquelet operation, demonstrating new intramedullary nail and cement spacer in place.

Post-operatively, the patient remained on intravenous antibiotics until post-operative day 4, when intraoperative cultures remained without growth. He was placed on 81 mg of Aspirin twice daily for deep venous thrombosis prophylaxis. Radiographs were obtained prior to hospital discharge, demonstrating the cement spacer's satisfactory position and the intramedullary nail [Fig. 4]. His drain was removed when wound drainage decreased to <30 cc/24 h on post-operative day 7. He was instructed to be toe-touch weight bearing with crutches until the second stage of his procedure. However, the patient was able to weight-bear with a single crutch within the first two weeks after surgery.

**Fig. 5:**
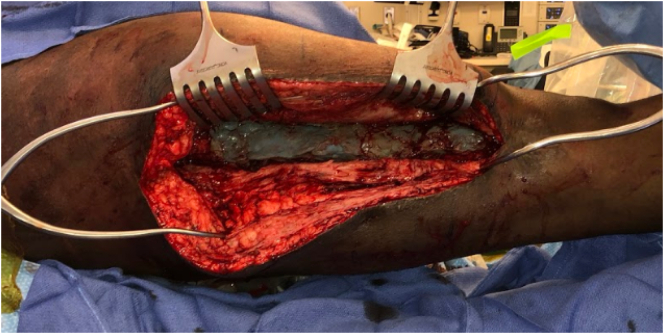
Demonstrating the cement spacer in place at stage 2 of the patient's Masquelet operation.

**Fig. 6:**
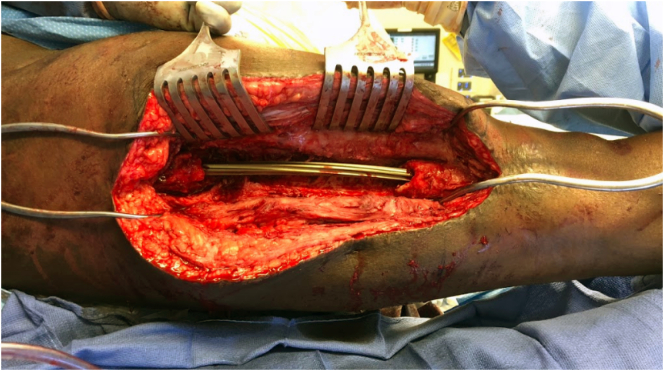
Demonstrating prior IM nail left in place after cement spacer removal during stage 2 of his operation.

**Fig. 7:**
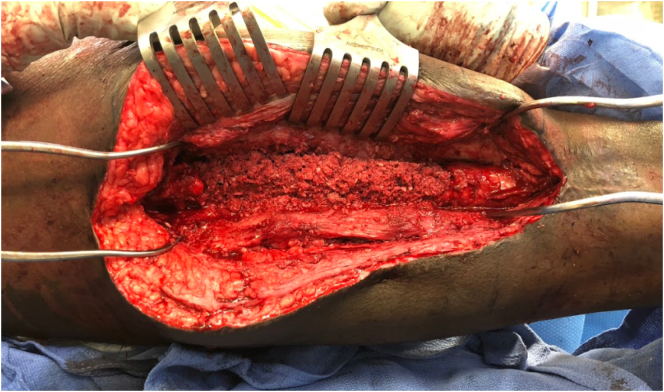
Bone graft mixture packed into membrane chamber prior to membrane closure during stage 2 of his operation.

Five weeks after the first stage, there was still no clinical or laboratory evidence of infection, and the second stage of his Masquelet operation was performed. He was placed on a fracture table with both legs prepped and draped. An incision was made through the previous scar, and the membrane chamber was accessed [Fig. 5]. New cultures were obtained, and the antibiotic cement spacer was removed while the prior intramedullary nail was left in place [Fig. 6]. The membrane chamber was thoroughly debrided of all scar, and fibrinous tissue and curretes were used to stimulate bleeding bone at the defect's proximal and distal ends. Next, 75 cc of autologous bone graft was taken from the contralateral femur with the RIA, and 60 cc of bone marrow aspirate was taken from the contralateral iliac crest. This was combined with 45 cc of allograft cancellous chips and 50 cc of ViviGen Cellular Bone Matrix (DepuySynthes; Johnson & Johnson Co. Inc., NJ). The autograft and allograft were loosely packed into the membrane chamber [Fig. 7]. Recombinant human bone morphogenetic protein-2 (INFUSE; medium pack; Medtronic Sofamor Danek, Minneapolis, MN) was then placed over the graft. The membrane was then tightly closed around the graft. Additional remaining bone marrow aspirate was injected with a needle through the membrane. No leakage was observed after injection, indicating a tight closure. The remaining wound was copiously irrigated and closed in a layered fashion.

Post-operatively, the patient was admitted to the hospital for observation and 48 h of prophylactic antibiotics. He was made non-weight bearing to his affected lower extremity, and a bone stimulator was started immediately. The intraoperative cultures from his second stage operation remained without growth.

**Fig. 8:**
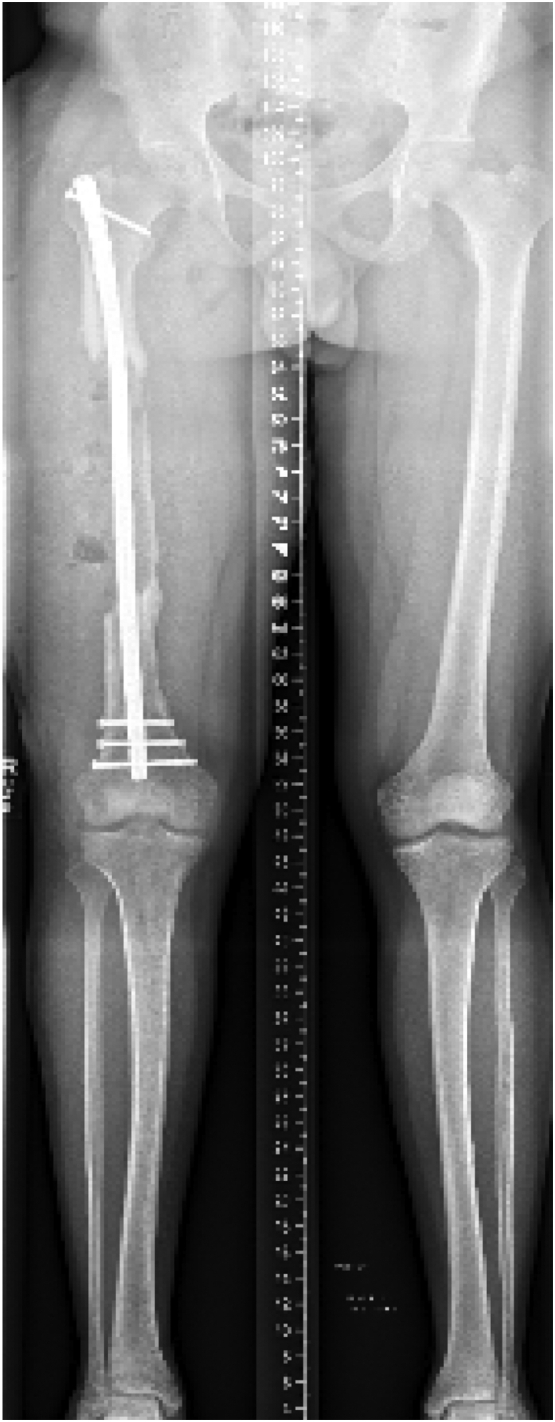
Standing alignment radiograph taken two weeks after second stage of his Masquelet operation, demonstrating intramedullary nail in place, with 20 cm defect with bone graft in place.

**Fig. 9:**
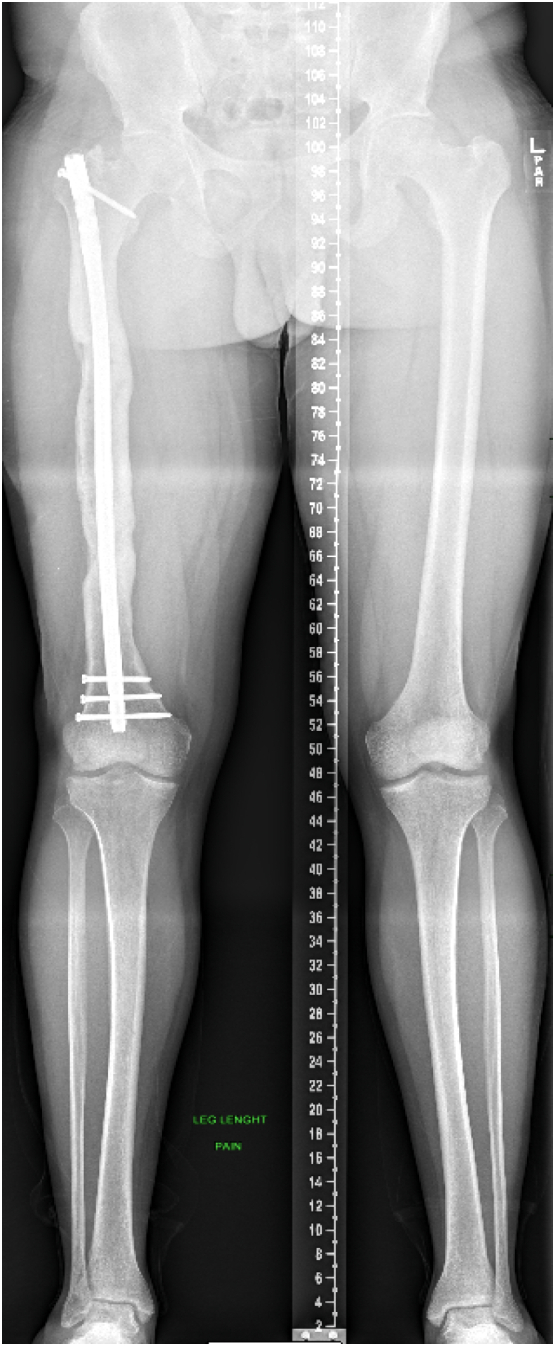
Standing full length femur image one year after completion of his Masqulet operation demonstrating complete union of bone defect and residual 2 cm leg length discrepancy.

**Fig. 10:**
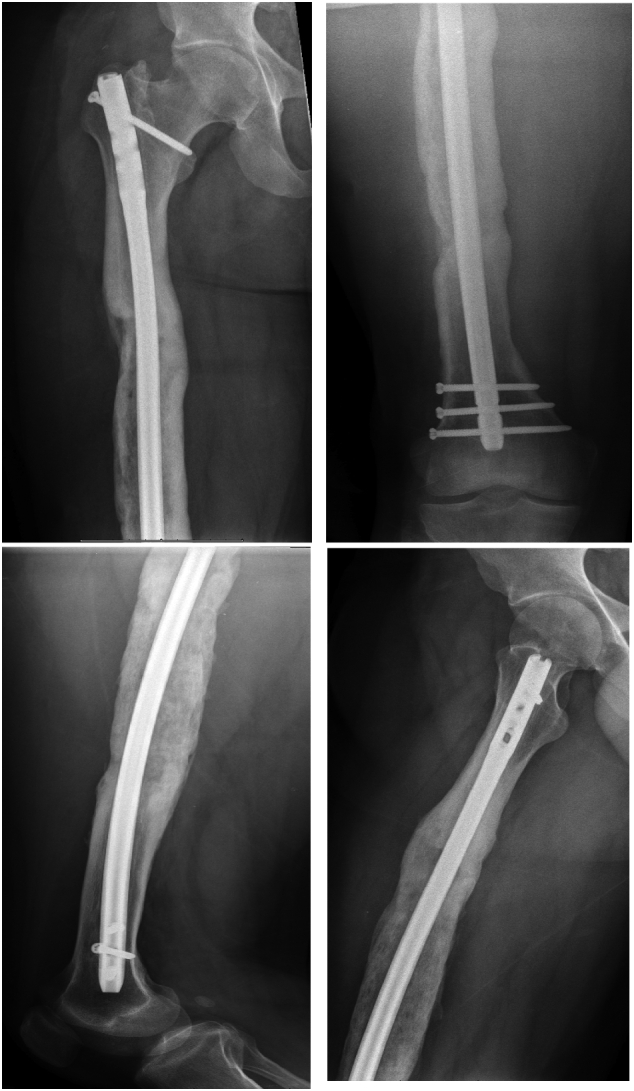
Full length femur images one year after completion of his Masqulet operation demonstrating complete union of bone defect.

The patient was followed clinically and radiographically throughout the next year to monitor his progress [Fig. 8]. Two weeks after his second stage Masquelet operation, he begun to weight-bear fully without crutches. At one year follow-up, the patient demonstrated clinical and radiographic union of his bone defect with a residual 2 cm leg length discrepancy [Fig. 9, Fig. 10]. At his two-year follow-up, radiographs continued to have a satisfactory appearance, and the patient endorsed overall significant improvement in quality of life and functionality. After the second stage of his operation, he returned to school, earned his paralegal certificate, and was the valedictorian of his class. He was noted to have continued right patellofemoral knee pain and required a shoe lift for his leg length discrepancy, which he did not routinely wear. Pain management was consulted. He underwent an inferior-medial genicular nerve block and nerve blocks of the sciatic and femoral nerves' sensory branches, which did not help. Two years after the second stage of his operation, his pain was well managed with 400 mg of Gabapentin 3 times daily and two tabs of 5/325 mg oxycodone-acetaminophen per day. Ultimately, his goal was to stop narcotics completely.

## 3 Discussion

Segmental bone loss due to trauma and subsequent infection is a challenging condition to manage. Satisfactory results have been demonstrated regarding treating these patients with the Masquelet technique [[1], [2], [3], [4], [5], [6], [7], [8]]. The Masquelet technique has most commonly been used to treat bone defects of the tibia, followed by the fibula and femur [3]. It has also been used to treat bone defects of the upper extremity, including the wrist and hand [1]. The mean size of defects treated with this technique reported in the literature is 5.53 cm [2]. To our knowledge, our case represents one of the most extensive femoral bone defects treated with the Masquelet technique. In Masquelet's original series, he reported the successful union of a 25 cm segmental defect of the tibia [3]. Stafford et al. described a 25 cm femoral defect and a 20 cm tibial defect treated successfully with the Masquelet technique [6]. Karger et al. described successful treatment with the Masquelet technique of a 23 cm defect in a tibia [5].

**Fig. 11:**
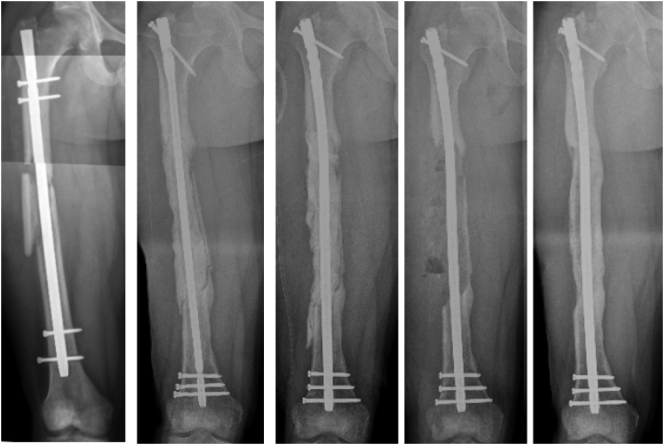
Radiographs demonstrating the patient's progression to complete union of the bone defect.

Our patient was interested in limb salvage surgery and declined bone transport. Given the significant size of his defect, we opted to treat him utilizing the Masquelet technique. He went on to have a successful union of his defect with associated increased subjective quality of life and functionality [Fig. 11].

The patient's initial cultures grew a multi-drug resistant species of enterococcus. This was thought to have been from his initial operation in Uganda, where there is a high antimicrobial resistance rate. This is due to numerous local factors, including the dispensing of over-the-counter antibiotics, inappropriate prescribing practices of antibiotics amongst medical professionals, lack of adequate laboratory services to provide targeted antibiotic therapy, as well as public perception of antibiotics able to treat almost any malady, infectious or not [9]. Our patient was appropriately managed with targeted antibiotic therapy and repeated surgical debridements, leading to his infection resolution before our successful Masquelet intervention. Pathogen-directed antibiotics were placed within the PMMA spacer. This local delivery of antibiotics via an antibiotic-impregnated PMMA spacer can achieve antibiotic concentration levels many times greater than the bacterial minimum inhibitory concentration, with negligible increases in serum or plasma levels and a small risk of systemic toxicity [1].

During the second stage of our patient's Masquelet operation, the intramedullary nail from the first stage was left in place, as leaving the prior intramedullary nail in place has been shown to provide satisfactory results [7]. We utilized a combination of bone graft types, including Reamer-Irrigator-Aspirator, iliac crest, as well as cancellous allograft chips, to provide adequate graft volume to fill our patient's bone defect. Of note, the RIA system results in significantly greater harvest volume, reduced harvest time, and improved donor site pain compared to iliac crest harvesting techniques [10]. We also added BMP-2 to improve its osteoconductivity and osteoinductivity, which has produced good results in prior series [[6], [7], [8]].

Despite our recommendations, our patient began fully weight-bearing at two weeks after his second stage operation, compared to 4–17 months until full weight bearing reported in prior series [1,5]. Given that our patient achieved a union of his bone defect, it is possible that patients treated with Masquelet technique and IM nailing can have weight-bearing status advanced sooner than previously thought if further studies support this outcome.

Compared to other bone reconstruction techniques effective for defects over 20 cm, such as bone transport, the Masquelet technique has the advantage that the healing time is independent of the defect length. Masquelet is generally better accepted and can be used in less compliant patients given that the prolonged use of an external frame is avoided [3]. While relatively new bone transport techniques utilizing internal lengthening nails are showing promising results, these techniques have limited utility for large defects due to limitations in the maximum amount of distraction that the available implants can achieve (i.e., the magnetically actuated lengthening PRECICE® nail (Nuvasive, Aliso Viego, CA, USA) allows for 8 cm of maximum distraction) [11].

In Summary, the Masquelet technique is a useful limb salvage treatment strategy for patients with segmental bone defects, including large defects of 20 cm in length.

Written informed consent was obtained from the patient for publication of this case report and accompanying images. A copy of the written consent is available for review by the Editor-in-Chief of this journal on request. This manuscript was written following the SCARE 2020 criteria [12].

## Sources of funding

None.

## Ethical approval

Ventura County Medical Center (VCMC), the site from which all data was gathered, does not require IRB approval for sufficiently anonymous case reports.

However, a consent was completed for this patient, which is available upon request.

## Consent

All possible identifying information has been removed to protect anonymity.

Informed consent was obtained from the patient for publication of this case report and accompanying images. A copy of the consent is available for review by the Editor-in-Chief of this journal on request.

## Research registration

None.

## Guarantor

Graal Diaz

## CRediT authorship contribution statement

Kyle Kubes, co-author.

Alex Friedman, co-author.

Casey Pyle, co-author.

Graal Diaz, co-author.

Damayea Hargett, primary attending surgeon (concept).

## Declaration of competing interest

This case report was presented in poster form at the American Osteopathic Academy of Orthopedic Surgeons (AOAO) annual meeting in October 2019.

Kyle Kubes, nothing to disclose.

Alex Friedman, nothing to disclose.

Casey Pyle, nothing to disclose.

Graal Diaz, nothing to disclose.

Damayea Hargett, nothing to disclose.

## References

[1] Taylor B.C., French B.G., Fowler T.T., Russell J., Poka A. (2012). Induced membrane technique for reconstruction to manage bone loss. J. Am. Acad. Orthop. Surg..

[2] Masquelet AC, Fitoussi F, Begue T, Muller GP. [Reconstruction of the long bones by the induced membrane and spongy autograft]. Ann. Chir. Plast. Esthet. 2000;45: 346–53. 10929461

[3] Morelli I., Drago L., George D., Gallazzi E., Scarponi S., Romano C. (2016). Masquelet technique: myth or reality? A systematic review and meta-analysis. Injury.

[4] Pelissier P., Masquelet A.C., Bareille R., Pelissier S.M., Amedee J. (2004). Induced membranes secrete growth factors including vascular and osteoinductive factors and could stimulate bone regeneration. J. Orthop. Res..

[5] Karger C., Kishi T., Schneider L., Fitoussi F., Masquelet A.C., French Society of Orthopaedic S (2012). Treatment of posttraumatic bone defects by the induced membrane technique. Orthop. Traumatol. Surg. Res..

[6] Stafford PR, Norris BL. Reamer-irrigator-aspirator bone graft and bi Masquelet technique for segmental bone defect nonunions: a review of 25 cases. Injury 2010;41(Suppl 2): S72–7. 10.1016/S0020-1383(10)70014-0 21144933

[7] Paul R. Stafford, Brent L. Norris. Reamer–irrigator–aspirator bone graft and bi Masquelet technique for segmental bone defect nonunions: a review of 25 cases. Inj. Int. J. Care Injured 41 (2010) S2, S72–S77. 10.1016/S0020-1383(10)70014-0 21144933

[8] Metsemakers W.J., Claes G., Terryn P.J., Belmans A., Hoekstra H., Nijs S. (2019, Feb). Reamer–irrigator–aspirator bone graft harvesting for treatment of segmental bone loss: analysis of defect volume as independent risk factor for failure. Eur. J. Trauma Emerg. Surg..

[9] UNAS, CDDEP, GARP-Uganda, Mpairwe Y., Wamala S. (2015). Antibiotic Resistance in Uganda: Situation Analysis and Recommendations (pp. 107). Kampala.

[10] Dawson J., Kiner D., Gardner W. (2014). The reamer-irrigator-aspirator as a device for harvesting bone graft compared with iliac crest bone graft: union rates and complications. J. Orthop. Trauma.

[11] Alrabai H.M., Gesheff M.G., Conway J.D. (2017). Use of internal lengthening nails in post-traumatic sequelae. Int. Orthop..

[12] Agha R.A., Franchi T., Sohrabi C., Mathew G., for the SCARE Group (2020). The SCARE 2020 guideline: updating consensus Surgical CAse Report (SCARE) guidelines. Int. J. Surg..

